# Human rights promotion and the ‘Geneva impasse’ in mental healthcare: scoping review

**DOI:** 10.1192/bjo.2023.50

**Published:** 2023-04-11

**Authors:** Bernadette McSherry, Piers Gooding, Yvette Maker

**Affiliations:** Melbourne Law School, University of Melbourne, Carlton, Australia; Faculty of Law, University of Tasmania, Hobart, Australia

**Keywords:** Human rights, Convention on the Rights of Persons with Disabilities, compulsory treatment, coercion, positive and negative rights

## Abstract

**Background:**

The World Psychiatric Association recently emphasised that the protection of human rights in mental healthcare was a ‘central concern’. This paper examines recent literature on human rights and mental healthcare.

**Aims:**

To (a) outline how international human rights law distinguishes between the protection and promotion of human rights; and (b) explore the literature on promoting human rights in mental healthcare which avoids what has been termed the ‘Geneva impasse’ between those who argue that compulsory care and treatment can never comply with human rights law and those who argue that they can if certain conditions are met.

**Method:**

The following doctrinal methodology was used: (a) identification and detailed analysis of international human rights conventions and commentaries; (b) identification of key literature on human rights and mental healthcare; and (c) critical analysis of key issues emerging from the literature.

**Results:**

Much of the literature on human rights and mental healthcare focuses on whether restrictions on compulsory care are required to meet the requirements of United Nations Conventions. There is an emerging literature identifying measures to promote the right to the enjoyment of the highest attainable standard of mental health.

**Conclusions:**

There has been a focus on protecting the rights to liberty and equality before the law for mental health patients. The nascent literature on promoting human rights in mental healthcare could mark a way forward beyond the ‘Geneva impasse’ that has dominated public debate in recent years.

The World Psychiatric Association's (WPA's) 2022 position statement^1^ calls for action to support the implementation of alternatives to coercion in mental healthcare and emphasises that the protection of human rights is a central concern. The statement reflects recent advances in international human rights law associated with the coming into force of the United Nations Convention on the Rights of Persons with Disabilities^2^ (CRPD). The CRPD provides a framework for the provision of mental healthcare that not only protects but also promotes human rights. It affirms the enjoyment by all people, including people with ‘mental health conditions and psychosocial disabilities’,^3^ of the human rights described in earlier conventions, such as the International Covenant on Civil and Political Rights^4^ (ICCPR) and the International Covenant on Economic, Social and Cultural Rights^5^ (ICESCR). The CRPD clarifies the obligations of states which are party to it (that is, states which have agreed to be bound by this treaty under international law) to promote and ensure the rights of persons with disabilities. As at 16 February 2023, 185 states and the European Union were party to the CRPD. This paper outlines recent themes in the literature on the CRPD and what they indicate for the future of mental healthcare.

## Method

We conducted a scoping review, which involved a broadly defined research question and the development of *post hoc* inclusion/exclusion criteria at study selection stage. We used a doctrinal approach, which entails research into the law and legal concepts as exemplified in legal documents detailing rules, principles, norms and interpretive guidelines. This involved: (a) identification and detailed analysis of international human rights conventions and commentaries; (b) identification of key literature on human rights and mental healthcare; and (c) critical analysis of key issues emerging from the literature.

Using the United Nations Treaty Collection, we identified several key United Nations treaties and associated commentaries, including the ICCPR,^4^ the ICESCR^5^ and the CRPD^2^ and the resolutions made in 2016 and 2017 by the United Nations’ Human Rights Council on Mental Health and Human Rights.^3,6^

A streamlined literature review was conducted to identify edited collections, books, articles and grey literature (such as reports, working papers and government documents) on mental health and human rights. Numerous search strings in multiple combinations were used in keyword fields, or abstract and title fields (where available in each database). The search strings used the terms ‘Convention on the Rights of Persons of Disabilities’ OR ‘CRPD’, combined with (using ‘AND’) combinations of the following: mental- (health, illness, disability or impairment); mental healthcare, psychiatric, psychiatry; disability, disabled, psychosocial disability; legal, legal developments; human rights, civil and political rights, economic social and cultural rights, ICCPR, ICESCR, right to health; Geneva impasse.

Searches were limited to English language results, published during a 10-year period from December 2012 to December 2022. Where these options were available, they were also limited to online full-text and peer-reviewed results.

The following research databases were used: PubMed (including MEDLINE, life science journals and online books); INFORMIT (encompassing AGIS, Health Collection, Health and Society Database); CINAHL Complete, Index to Legal Periodicals; PROQUEST (which includes the Health and Medical Collection and Psychology Database); Science Direct Journals; SSRN; Google Scholar; and LegalTrac. Despite the restrictive search terms used, Google Scholar results amounted to 14 200 papers and we decided to omit these on the basis that they were too numerous to be included.

A total of 482 results were identified based on the search terms. Fifty-six of these articles were duplicates and removed, leaving a total of 426 unique results (Table 1).

**Table 1:** Number of results by search database

Database	Retrievals, *n*
Informit	30
LegalTrac	9
Proquest	57
Pubmed	147
ScienceDirect	215
SSRN	24
Preliminary total	482
Duplicates removed	−56
Revised total	426

After an initial scan of the literature, we included those results with a central focus on the CRPD and which dealt with the themes of (a) protecting or promoting human rights, or (b) human rights and disability, or (c) human rights and mental healthcare, or (d) the right to the enjoyment of the highest attainable standard of mental health.

We excluded the results that (a) did not contain the term ‘CRPD’ or ‘Convention on the Rights of Persons with Disabilities’ in the title, abstract or keywords, or (b) did not focus on the area of ‘mental health’ or ‘disability’, defined in the study to include the terms ‘mental healthcare’, ‘mental illness’, ‘mental disorder’, ‘psychosocial disorder’, ‘psychiatric disorder’, ‘psychological disorder’, ‘psychiatry’ or ‘psychology’.

We excluded 325 results for not meeting the study criteria, leaving 101 results which we analysed in terms of key themes dealing with protecting human rights in mental healthcare and those referring to promoting human rights, particularly the right to the enjoyment of the highest attainable standard of mental health.

## Results

International treaties establish that human rights are inherent to all human beings, regardless of status. There has been a persistent view in the literature that there are two categories of rights. One category refers to civil and political rights (set out in the ICCPR), such as the rights to liberty and equality, which give rise to ‘negative’ obligations whereby the state must refrain from taking actions that infringe those rights. The other category refers to economic, social and cultural rights (set out in the ICESCR), which give rise to ‘positive’ obligations on the state to allocate resources to achieve the enjoyment of those rights. The ICCPR requires states to ‘respect’ and ‘ensure’ civil and political rights, obligations assumed to be capable of immediate implementation. In comparison, there is a general obligation in the ICESCR requiring states to take steps to achieve the progressive realisation of economic, social and cultural rights.

This dichotomy between negative and positive rights has been the subject of much questioning and debate, including in the literature about mental health law. What is significant about the CRPD is that it combines both sets of rights into the one treaty and challenges the notion that rights can be divided into categories, instead stressing their interconnectedness.^7^ These rights include the right to life (Article 10), the right to equal recognition before the law (Article 12), the right to liberty and security of the person (Article 14), the right to respect for physical and mental integrity (Article 17), the right to live in the community (Article 19), the right to education (Article 24) and the right to enjoyment of the highest attainable standard of health without discrimination on the basis of disability (Article 25).

Article 19, which is entitled ‘living independently and being included in the community’, provides a good example of the interconnectedness of negative and positive rights. The first paragraph of Article 19 deals with persons with disabilities having the opportunity to choose their place of residence and where and with whom they live. This has been interpreted by the Committee on the Rights of Persons with Disabilities (the Committee set up to monitor implementation of the CRPD) as a civil right which is subject to immediate recognition.^8^ The next two paragraphs, which deal with access to services to support living and inclusion in the community, as well as access to community services and facilities on an equal basis with others, have been interpreted as social rights, subject to progressive realisation.^8^

In 2016, the United Nations’ Human Rights Council passed a resolution on mental health and human rights^6^ which recognised ‘the need to protect, promote and respect all human rights in the global response to mental health-related issues, and stressing that mental health and community services should integrate a human rights perspective so as to avoid any harm to persons using them’ (p. 2). On 28 September 2017, the Human Rights Council approved a further resolution on mental health and human rights.^3^ Although these resolutions are not legally binding under international law, they signal a strong consensus from United Nations’ member states.

Paragraph 5 in this latter resolution recognises that it is necessary for states to ‘take active steps to fully integrate a human rights perspective into mental health and community services’. It also refers to the need to promote the right of everyone ‘to full inclusion and effective participation in society’. These words echo Article 3 of the CRPD, which includes as a general principle the ‘[f]ull and effective participation and inclusion in society’ of persons with disabilities, including persons with mental health conditions and psychosocial disabilities.

The United Nations Special Rapporteur on the Rights of Persons with Disabilities, an independent expert who provides advice to the United Nations on disability human rights, is currently exploring options for the societal participation of those with psychosocial disabilities, including through supported decision-making mechanisms and independent living services designed and run by persons with disabilities.^9,10^

Much of the literature about human rights and mental healthcare has centred on the meaning of Article 12 (the right to equal recognition before the law), the existence of compulsory treatment and how human rights can be integrated into services based on treatment without consent. Diverging viewpoints on this matter have emerged between two United Nations human rights ‘treaty bodies’, which are comprised of committees of independent experts that monitor implementation of the core international human rights treaties, such as the CRPD. Martin & Gurbai^11^ have referred to a ‘Geneva impasse’ that has emerged, whereby the United Nations Committee on the Rights of Persons with Disabilities^12^ and certain commentators, such as Minkowitz,^13^ have interpreted the CRPD to mean that legislation enabling treatment without consent should be abolished, whereas the Human Rights Committee^14^ and other commentators, such as Dawson^15^ and Wilson,^16^ have interpreted it as allowing for processes for substituted decision-making, but only in exceptional circumstances, such as where there is a risk of serious harm to self or others.

There have, however, also been commentaries on the CRPD more generally and on the importance of the role of people with disabilities and their representative organisations working to develop services that people with disabilities, including people with mental health conditions and psychosocial disabilities, want.^17^ According to Dainius Pūras, the former United Nations Special Rapporteur on the right to the highest quality physical and mental health:

‘participation of persons with mental health conditions, including persons with disabilities, in the planning, monitoring and evaluation of services, in system strengthening and in research, is now more widely recognized as a way to improve the quality, accessibility and availability of services and the strengthening of mental health systems’.^18^

Bartlett^19^ makes the additional point that the CRPD as a whole requires that ‘as is the case with other citizens, services are to be offered, not imposed’.

Some literature avoids the ‘Geneva impasse’ by focusing on what the right to the highest attainable standard of health means for mental healthcare rather than focusing on which actions might breach the rights set out in the CRPD.^20,21^ Attention is turning to how practical measures such as psychiatric advance directives,^22,23^ different models of supported decision-making^24^ and human rights training for mental health service providers, patients, families and carers^25^ may assist in promoting human rights.

There is also a developing literature on the need for states to promote good mental health through appropriate allocation of resources and related ‘positive’ human rights.^26,27^ The main point of consensus is that coercion in mental healthcare can and should be reduced^1,9,10,28^ and the range of voluntary options for support be expanded.^29–32^

## Discussion

Human rights debates about mental healthcare have traditionally focused on the rights to liberty and autonomy in relation to the compulsory treatment of persons with psychosocial disabilities. Commentaries on the CRPD have largely focused on what the right to equal protection before the law means for the existence of compulsory treatment schemes and whether such schemes should be abolished or reformed. Breaking through the so-called ‘Geneva impasse’ might seem difficult at first glance. However, although disagreements about the legitimacy of compulsory treatment and coercive practices persist, there is optimism that these practices can be reduced and attention is turning to how best to achieve this. The Council of Europe, for example, has established a compendium of ‘good practices’ to promote voluntary care and treatment.^33^ European Cooperation in Science and Technology (COST) has also funded a research network called FOSTREN, which is dedicated to fostering and strengthening approaches to reducing coercion in European mental health services.^34^ The importance of implementing a rights-based approach to mental healthcare is not only the subject of commentary on the CRPD but has also been recognised in legislation. For example, section 12(c) of the Mental Health and Wellbeing Act 2022 in the state of Victoria, Australia, sets out that an objective of the legislation is ‘to provide for comprehensive, compassionate, safe and high-quality mental health and wellbeing services that promote the health and wellbeing of people living with mental illness or psychological distress’. Section 12(e) states that a further objective is ‘to protect and promote the human rights and dignity of people living with mental illness by providing them with assessment and treatment in the least restrictive way possible in the circumstances’.

The right to the enjoyment of the highest attainable standard of mental health has been recognised and discussed as an element of the broader right to health established in foundational human rights instruments such as the ICESCR. Although economic, social and cultural rights have been distinguished in the past on the basis that they require progressive rather than immediate realisation, that distinction is challenged by the blending of ‘positive’ and ‘negative’ rights within the CRPD. States that have ratified the CRPD have obligations to promote economic, social and cultural rights, including the right to employment, the right to social security and the right to an adequate standard of living, all of which are associated with good mental health. Measures aimed at promoting these rights may indirectly reduce compulsory care and treatment, although this was not a focus in the literature analysed.

It should be noted that our analysis of the literature was limited to English language material. While the United Nations has six official languages, English is one of the two working languages of the United Nations Secretariat and most relevant ‘international’ journals are published in English. There is a need, however, to examine papers in languages other than English in order to discover any differences in focus.

In 2017, the WPA–*Lancet Psychiatry* Commission stated^35^ that ‘compulsory treatment cannot be sensibly divorced from the provision of appropriate services, most required by the CRPD, that people want to use’. An emphasis on all categories of human rights, be they civil, political, economic, social or cultural, would seem to provide a framework for how best to provide the mental healthcare and treatment that persons with mental health conditions and psychosocial disabilities need. That way, the ‘Geneva impasse’ may become the ‘Geneva pathway’ to promoting human rights and high-quality mental healthcare.

## Data Availability

Data availability is not applicable to this paper as no new data were created or analysed in this scoping review.
